# The impacts of economic hardship and family long-term care needs on adolescent depression and aggressive behaviors through family relational mechanisms: from the perspective of family stress model

**DOI:** 10.1186/s13034-025-01014-z

**Published:** 2025-12-24

**Authors:** Ji-Kang Chen, Chaoyue Wu

**Affiliations:** 1 The Chinese University of Hong Kong, Shatin,, Hong Kong; 2 University of California, Los Angeles, USA

**Keywords:** Family stress model, Adolescent depression, Aggressive behavior, Economic hardship, Long-term care, Parental conflict, Parent-child relationship

## Abstract

**Background:**

Even though the theoretical rationale for extending Family Stress Model (FSM) to include family long-term care needs is substantiated, it has seldom been empirically integrated within the FSM framework, especially in Asian cultural settings. The current study longitudinally investigated how family stressors (economic hardship and long-term care needs) affect adolescent depression and aggressive behaviors through family relational mechanisms (parental conflict and parent-child relationship) and how these relationships differ by gender in mainland China.

**Methods:**

This study employed structural equation modeling to analyze two-wave longitudinal national data from the China Education Panel Survey. The sample consisted of 9,433 student-parent pairs across 112 schools in 28 county-level units throughout mainland China who participated in both waves.

**Results:**

The results of serial structural equation modeling indicated that family economic hardship in Wave 1 did not significantly predict adolescent depression and aggressive behaviors in Wave 2 directly. However, it showed significant indirect effects on depression and aggressive behaviors through parental conflict in Wave 1 and parent-child relationship in Wave 2. Family long-term care needs in Wave 1 directly predicted adolescent depression in Wave 2 and indirectly predicted both depression and aggressive behaviors in Wave 2 through parental conflict. The overall model explained 10.4% of variance in depression and 6.1% in aggressive behaviors. Multi-group analysis revealed that the theoretical model of this study was applied to both genders. However, female adolescents showed stronger sensitivity to family stressors and relational processes. The model accounts for 13.6% of variance in depression and 10.8% in aggressive behaviors for females, compared to 9.2% and 4.4% for males, respectively.

**Conclusions:**

The findings suggest that economic hardship is a stronger family stressor than long-term care needs, and parental conflict serves as a more significant mediator than parent–child relationship quality in predicting adolescent depression and aggressive behaviors in Chinese contexts. These results highlight the importance of developing stressor-specific and culturally sensitive family interventions that interparental conflicts to effectively reduce the negative impacts of family stress on adolescent mental and behavioral health. Additionally, gender-sensitive interventions may be particularly beneficial due to stronger family stress effects on relational outcomes, depression, and aggressive behaviors among female adolescents.

## Introduction

 Depression and aggressive behaviors have become significant global public health issues, affecting both immediate and long-term well-being among adolescents [1–3]. Family contexts play a particularly essential role in shaping adolescent psychological and behavioral health [4, 5]. The Family Stress Model (FSM) provides a theoretical framework explaining how various family stressors influence adolescent development through relational mechanisms such as interparental conflict, disrupted parenting practices, and impaired parent–child relationships [6, 7]. An increasing number of empirical studies have supported FSM, demonstrating that family stressors such as economic hardship negatively affect adolescent outcomes through increases in parental conflict and the deterioration of parent–child relationship quality [8–12].

However, recent literature has emphasized the need for broadening FSM’s scope to consider additional family stressors that similarly disrupt family functioning and adolescent outcomes [13]. One emerging but understudied stressor is family long-term care needs for chronically ill family members. Such caregiving demands might substantially amplify economic burdens by reducing household income, increasing medical expenses, and diminishing parental employment opportunities [14–16]. Additionally, caregiving responsibilities impose emotional and relational strains, potentially increasing interparental conflict and negatively impacting the quality of parent–child relationships, which may influence adolescent mental health and behaviors [13].

Even though the theoretical rationale for extending FSM to include family long-term care needs is compelling, family long-term care needs have seldom been empirically integrated within the FSM framework. Moreover, FSM research has primarily been conducted in Western, developed contexts, raising critical concerns regarding its generalizability to non-Western societies and developing countries. To address these gaps, the current study uses a nationally representative Chinese sample to extend FSM by examining family economic hardship and family long-term needs as stressors. Employing longitudinal, multi-informant data, this study tests the FSM’s applicability to diverse family financial stressors in an Asian cultural context.

## Literature review

### Family economic hardship and adolescent outcomes

Family economic hardship is a well-documented risk factor for adolescent mental health and behavioral outcomes [17–19]. Studies have consistently revealed significant associations between family economic status and adolescent psychological distress and behavioral problems, with lower family socio-economic status (SES) adolescents showing higher rates of depression, anxiety, aggression, and delinquency behaviors [20–23]. For example, McLaughlin et al. [24] analyzed data from the National Comorbidity Survey Adolescent Supplement and found that adolescents in the lowest SES quartile were more likely to develop major mental health issues and conduct disorder compared to those in the highest income quartile. However, a few studies have found weak or non-significant direct associations when controlling for other contextual variables [25, 26], suggesting that family income may influence adolescent development indirectly. In addition, studies relying on adolescent-reported economic data may introduce potential inaccuracies given adolescents’ typically limited awareness of household financial details [27–29]. It may also contribute to variability in findings across studies.

### Family long-term care needs and adolescent outcomes

Family long-term care needs, defined as the ongoing care required for family members with chronic illness or health conditions, represent a significant but understudied family stressor that may influence adolescent depression and aggressive behaviors [15, 30]. These situations arise when family members have serious medical conditions, disabilities, or chronic illnesses that require ongoing medical attention, supervision, or assistance with daily activities [31–33].

Unlike general economic hardship, the financial stress associated with long-term care needs often emerges from a combination of extra family costs [31–33]. Families may face increased medical expenses from caregiving supplies and transportation costs, while also experiencing reduced income due to caregivers cutting back on work or foregoing job opportunities [34]. These long-term and unpredictable expenses may impose chronic financial, emotional, and relational strains on families [32, 33], which may negatively affect adolescent well-being [15, 30].

However, empirical research on how family long-term care needs affect adolescent mental health and behavioral outcomes is limited. Most studies have focused on adult caregivers or elderly populations [35–37], with adolescents largely overlooked as affected family members. Among the few studies examining the impacts of long-term care needs on children and adolescents, most have primarily investigated extreme cases, such as childhood cancer or severe disability [38], leaving the broader impact of caregiving responsibilities on adolescents underexplored [39]. Adolescents may be indirectly affected by shifts in household dynamics, reduced parental attention, or emotional strain within the family [40]. In addition, some studies have relied on small and clinical samples and cross-sectional designs, limiting the generalizability and longitudinal understanding of their findings.

### Parental conflict and parent–child relationship as mediators

The FSM posits that families function as interconnected systems where stress in one domain creates spillover effects throughout the entire family structure [41, 42]. The model identifies a sequential mediational process whereby family stressors disrupt the quality of marital relationships and increase parental conflict. Deteriorated parental interactions then spill over into parent–child relationships, as stressed and conflicted parents become less emotionally available and more inconsistent in their parenting. These disrupted family relational processes serve as the primary mechanisms through which original stressors affect adolescent adjustment outcomes [7]. Extensive empirical research has applied the FSM to examine the direct and indirect impacts of family stressors on adolescent development. Studies consistently demonstrate that family relational processes mediate the associations between family economic stressors and adolescent mental and behavioral health [7, 8, 10]. Masarik and Conger’s [13] comprehensive review found that family relational processes consistently mediated economic hardship effects across studies, with particularly strong evidence for the sequential pathway from economic stress to marital problems to parent–child relationship difficulties.

While the FSM has been supported by substantial empirical evidence, several important gaps in the literature may limit its theoretical scope and broader applicability. Most FSM research has largely focused on economic hardship while overlooking other significant family stressors that may impact adolescent development through similar pathways, such as family long-term care needs [13].

Testing the FSM in Chinese society is particularly important, given China’s status as a developing country with income inequality and limited social welfare systems, which may intensify FSM pathways. Despite recent long-term care insurance pilot programs, the majority of care responsibility still falls on family members, especially for economically disadvantaged families with limited access to formal services [43, 44]. Families need to bear greater responsibility for managing both economic hardship and caregiving needs with limited external support [44], which could deteriorate family relations and increase the risk of adolescent developmental problems.

In contrast, some researchers have suggested that the Chinese culture and norms may mitigate the negative impacts of long-term care needs. China has been characterized by collectivism cultural values, including familism and filial piety, which emphasize strong moral obligations to care for family members and preserve family harmony [45, 46]. Therefore, the application of the FSM to caregiving contexts may be buffered in Chinese societies compared to Western contexts, where caregiving is often conceptualized primarily as an external stressor rather than a normative family obligation. However, empirical evidence on the FSM’s applicability to long-term care needs in Chinese contexts remains limited.

It has also been argued that the relationships between family stressors, family relational mechanisms, and adolescent outcomes may differ by gender within the Family Stress Model framework [8, 10]. For example, recent studies have indicated that economic hardship has stronger effects on family processes among families with female adolescents [23, 47] and that the strength of the link between parental conflict and adolescent adjustment varies by gender, with girls showing greater vulnerability to interparental conflict [48, 49]. Additionally, traditional Chinese gender socialization may create differential exposure to family caregiving responsibilities, with girls more likely to be involved in or aware of family care dynamics [50, 51]. In contrast, other recent studies suggest the interrelationships may be similar across gender, showing insignificant gender differences in the association between family economic stress and adolescent outcomes and between family conflict and youth behavioral problems [11, 12]. Given these mixed findings and the cultural context of traditional Chinese gender roles in family caregiving, examining potential gender differences in Family Stress Model pathways represents an important direction for understanding how family stressors differentially affect adolescent development in Chinese contexts.

### The present study

This study aims to address FSM research gaps by examining how economic hardship and family long-term care needs impact adolescent depression and aggressive behaviors through parental conflict and parent–child relationship quality using data from a national longitudinal sample in China and how these relationships differ by gender. Specifically, the hypotheses are:

#### H1

Family economic hardship and family long-term care needs at Wave 1 are positively associated with parental conflict at Wave 1.

#### H2

Parental conflict at Wave 1 negatively predicts parent–child relationship quality at Wave 2.

#### H3

Parent–child relationship at Wave 2 is negatively associated with adolescent depression and aggressive behaviors at Wave 2.

#### H4

Parental conflict and parent–child relationship quality serially mediate the relationships between family stressors (economic hardship, family long-term care needs) and adolescent outcomes (depression, aggression).

#### H5

The model applies to both genders; however, the strength of the relationships may differ by gender. Specifically, female adolescents tend to show stronger associations between family stressors and family relational mechanisms, as well as between family relational mechanisms and adolescent outcomes, compared to male adolescents.

## Methods

### Data and procedure

This study conducted a secondary data analysis utilizing samples from the China Education Panel Survey (CEPS) [52], a large-scale, nationally representative, longitudinal survey aimed at documenting educational processes and transitions throughout students’ academic trajectories. It administers five different questionnaires to collect comprehensive data from multiple perspectives, including students, parents, homeroom teachers (teachers who not only provide subject instruction but also take primary responsibility for the overall management, guidance, and well-being of students in their class), main subject teachers who are not the homeroom teacher, and school administrators.

The CEPS employed a stratified, multistage sampling design using probability proportional to size to obtain a nationally representative, school-based sample. The survey included approximately 20,000 students from 438 classrooms across 112 schools in 28 county-level units throughout mainland China. The baseline survey (Wave 1), conducted during the 2013–2014 academic year, included 10,279 seventh-grade students and their parents (52.7%) and 9,208 ninth-grade students and their parents (47.3%). The second wave of data collection (Wave 2), conducted during the 2014–2015 academic year, followed the seventh-grade cohort and their parents as the students advanced to eighth grade. Of the original seventh-grade sample, 9,449 student–parent pairs were successfully followed in Wave 2, resulting in a follow-up rate of 91.9%.

This study utilized data from both students and their parents across both waves. The student survey gathered information about family relationship dynamics as well as psychological and behavioral outcomes, while parent survey questionnaires focused on household economic conditions and family long-term care needs. The analytic sample consisted of student–parent pairs who participated in both waves and completed survey questions on key study variables in this study. After excluding cases with missing data, the final sample included 9,433 student–parent pairs.

The CEPS data are publicly available for academic research purposes.

### Measures

#### Independent variables

*Family Economic Hardship* Family economic hardship was assessed using two parent-reported items from the Wave 1 parent questionnaire. Parents responded to: (a) “How is the financial condition of your family at present?” originally rated on a 5-point scale from 1 = very poor to 5 = very rich; and (b) “Which is the income level of your family in your community?” originally rated on a 5-point scale from 1 = very low to 5 = very high. To facilitate interpretation where higher scores indicate greater economic hardship, both items were reverse-coded (1 = very rich/high, 2 = rich/high, 3 = average, 4 = poor/low, 5 = very poor/very low). Cronbach’s alpha for these two items was 0.78.

*Family Long-term Care Needs* Family long-term care needs were measured by a single dichotomous item from the Wave 1 parent questionnaire: “Anyone in your family needs long-term care due to illness or mobility problems?” Response options were yes (coded as 1) or no (coded as 0).

#### Mediators

*Parental Conflict* Parental conflict was assessed using a single item from the Wave 2 student questionnaire: “My parents quarrel a lot - Do you agree with the following statement?” Students responded with yes (coded as 1) or no (coded as 0).

*Parent–Child Relationship* The quality of parent–child relationships was measured using two items from the Wave 2 student questionnaire: (a) “How is the general relationship between you and your father?” and (b) “How is the general relationship between you and your mother?” Both items used a 3-point scale (1 = not close, 2 = average, 3 = very close). Higher scores indicated closer relationships. Cronbach’s alpha for these two items was 0.61.

#### Outcome variables

*Depression* Depressive symptoms were assessed using six items from the Wave 2 student questionnaire, adapted from the Center for Epidemiologic Studies Depression Scale [53]. Students reported how frequently they experienced the following feelings in the past week: (a) Feeling blue, (b) Too depressed to focus on anything, (c) Unhappy, (d) Not enjoying life, (e) Having no passion to do anything, and (f) Sad, sorrowful. Responses were provided on a 5-point frequency scale (1 = never, 2 = seldom, 3 = sometimes, 4 = often, 5 = always). Higher scores indicated more severe depressive symptoms. Cronbach’s alpha for this scale was 0.92.

*Aggression Behaviors* Aggressive behaviors were measured using three items from the Wave 2 student questionnaire. Students reported the frequency of: (a) Cursing or saying swearwords, (b) Quarreling with others, and (c) Having a fight with others. Each behavior was rated on a 5-point frequency scale (1 = never, 2 = seldom, 3 = sometimes, 4 = often, 5 = always). Higher scores indicated more frequent aggressive behaviors. Cronbach’s alpha for this scale was 0.74.

#### Analysis plan

Demographics and descriptive statistics, including means, standard deviations, frequencies, and percentages, were calculated for study variables using SPSS 24. Bivariate correlations were examined to assess preliminary associations among variables. Structural equation modeling was conducted using Mplus X to test the hypothesized mediation models. The models examined whether parental conflict and parent–child relationship quality at Wave 2 mediated the associations between family economic hardship and long-term care needs at Wave 1 and psychological/behavioral outcomes at Wave 2.

Model fit was evaluated using multiple indices, including the chi-square test, comparative fit index (CFI), Tucker–Lewis index (TLI), root mean square error of approximation (RMSEA), and standardized root mean square residual (SRMR) [54]. A non-significant chi-square value (*p* > .05) indicates good model fit; however, this test is highly sensitive to large sample sizes and often yields significant results even when the model fits well [55]. For the CFI and TLI, values above 0.90 indicate acceptable fit, while values above 0.95 reflect a good fit. RMSEA values below 0.06 and SRMR values below 0.08 are also indicative of good model fit [54].

## Results

Sample demographics are shown in Table 1. Descriptive statistics (means and standard deviations) and the results of the bivariate correlations between major variables are shown in Table 2.

**Table 1:** Sample Demographics (*n* = 9449)

Variables	*n*	%
Gender		
Female	4441	51.2
Male	4838	47.0
Only child status		
Only child	4125	43.7
Have (a) sibling(s)	5181	54.8
Type of *Hukou*		
Agricultural Hukou	4546	48.1
Non-Agricultural Hukou	2349	24.9
Residential Hukou	1942	20.6
I have no Hukou	29	0.3
Family financial condition		
Very rich	26	0.3
Somewhat rich	491	5.2
Moderate	6676	70.7
Somewhat poor	1593	16.9
Very poor	356	3.8
Father’s Education background		
Elementary school	1227	13.0
Junior high school	3969	42.0
High school or equivalent degree	2129	26.2
Undergraduate or equivalent degree	1363	14.4
Graduate-level degree	132	1.4
Mother’s Education background		
Elementary school	1734	18.4
Junior high school	3818	40.4
High school or equivalent degree	2482	22.6
Undergraduate or equivalent degree	1165	12.3
Graduate-level degree	81	0.9

Agricultural Hukou refers to rural Hukou, a record that identifies a person as a rural resident.Non-agricultural Hukou refers to urban Hukou. Residential Hukou is a general record of residenceassigned to all residents of a certain region, regardless of rural or urban background

**Table 2:** Descriptive statistics and longitudinal correlations matrix of study *Variables*

Variables (M ± SD or %)	1	2	3	4	5
1. Family Economic Hardship (3.26 ± 0.59)					
2. Family Long-Term Care Needs (12.6%)	0.220**				
3. Parental Conflict (9.5%)	0.073**	0.049**			
4. Parent-Child Relationship (2.60 ± 0.46)	− 0.073**	− 0.019	− 0.184**		
5. Depression (2.16 ± 0.91)	0.068**	0.045**	0.142**	− 0.245**	
6. Aggressive Behavior (1.80 ± 0.70)	0.034**	0.028**	0.108**	− 0.186**	0.283**

**Correlation is significant at the 0.01 level (2-tailed)

### Mediation analysis

**Fig. 1:**
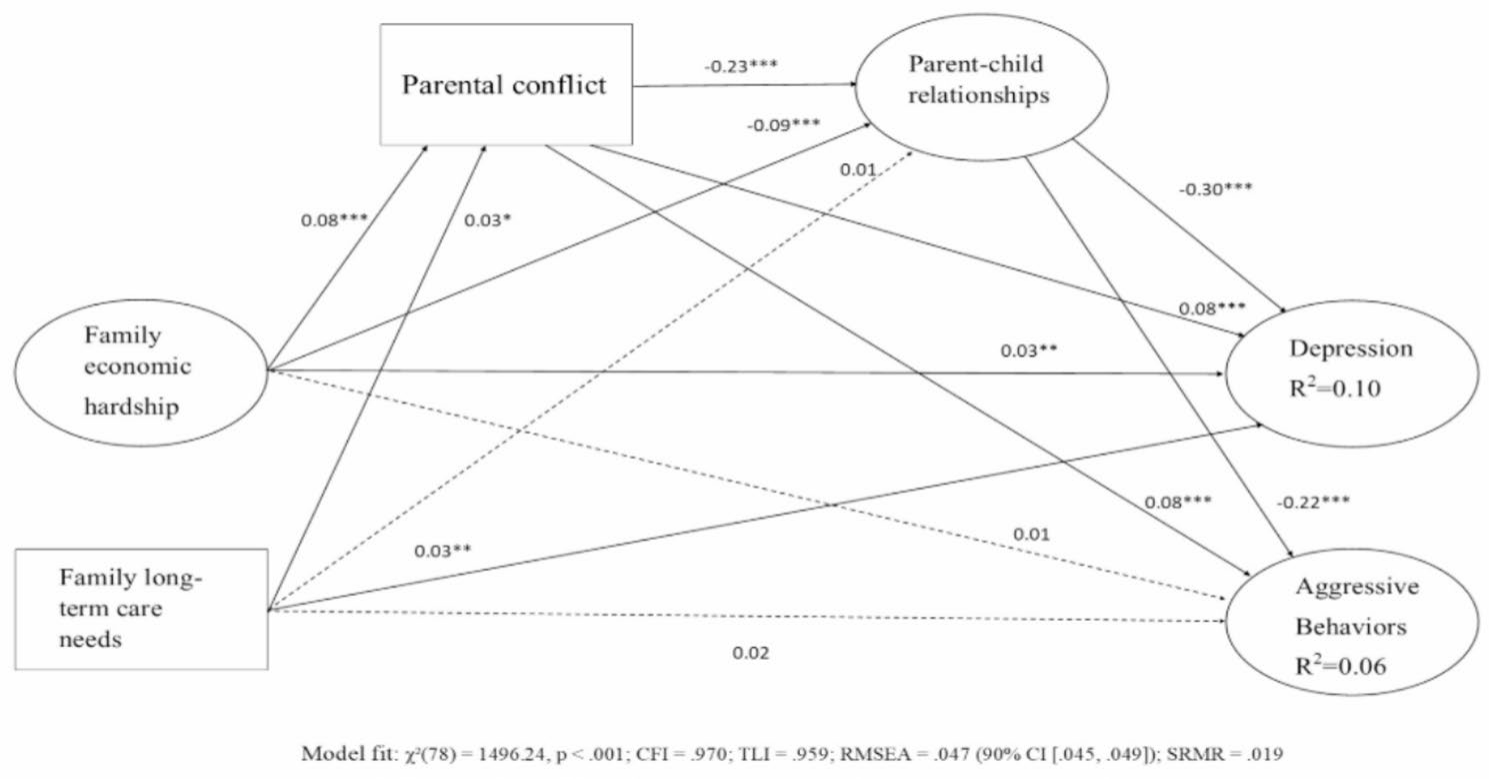
The serial longitudinal mediation model of the impacts of economic hardship and family long-term care needs on adolescent depression and aggressive behaviors through parental conflict and parent-child relationship quality *p < .05, **p < .01, ***p < .001

The hypothesized structural equation model demonstrated a good fit to the data: χ²(78) = 1496.24, *p* < .001; CFI = 0.970; TLI = 0.959; RMSEA = 0.047 (90% CI [0.045, 0.049]); SRMR = 0.019. As shown in Fig. 1, family economic hardship was directly associated with adolescent depression (β = 0.03, 95% CI [0.01, 0.06], *p* < .05). The total direct effect from family economic hardship to adolescent depression is 0.03 (95% CI [0.01, 0.01], *p* < .001). In addition, it indirectly influenced depression through increased parental conflict (β = 0.08, 95% CI [0.05, 0.11], *p* < .001), which was associated with poorer parent–child relationship quality (β = −0.23, 95% CI [-0.26, − 0.20], *p* < .001), and in turn predicted higher depressive symptoms (β = −0.30, 95% CI − 0.33, − 0.26], *p* < .001). Significant indirect effects were observed through both the single mediation via parental conflict (β = 0.01, 95% CI [0.01, 0.02], *p* < .001) and parent–child relationship (β = 0.03, 95% CI [0.03, 0.07], *p* < .001), as well as the sequential mediation via both parental conflict and parent–child relationship (β = 0.01, 95% CI [0.01, 0.02], *p* < .001), resulting in a total indirect effect of β = 0.04 (95% CI [0.01, 0.01], *p* < .001). Taken together, the total effect from family economic hardship to depression through parental conflict and parent-child relationships was 0.07 (95% CI [0.01, 0.01], *p* < .001)..

**Fig. 2:**
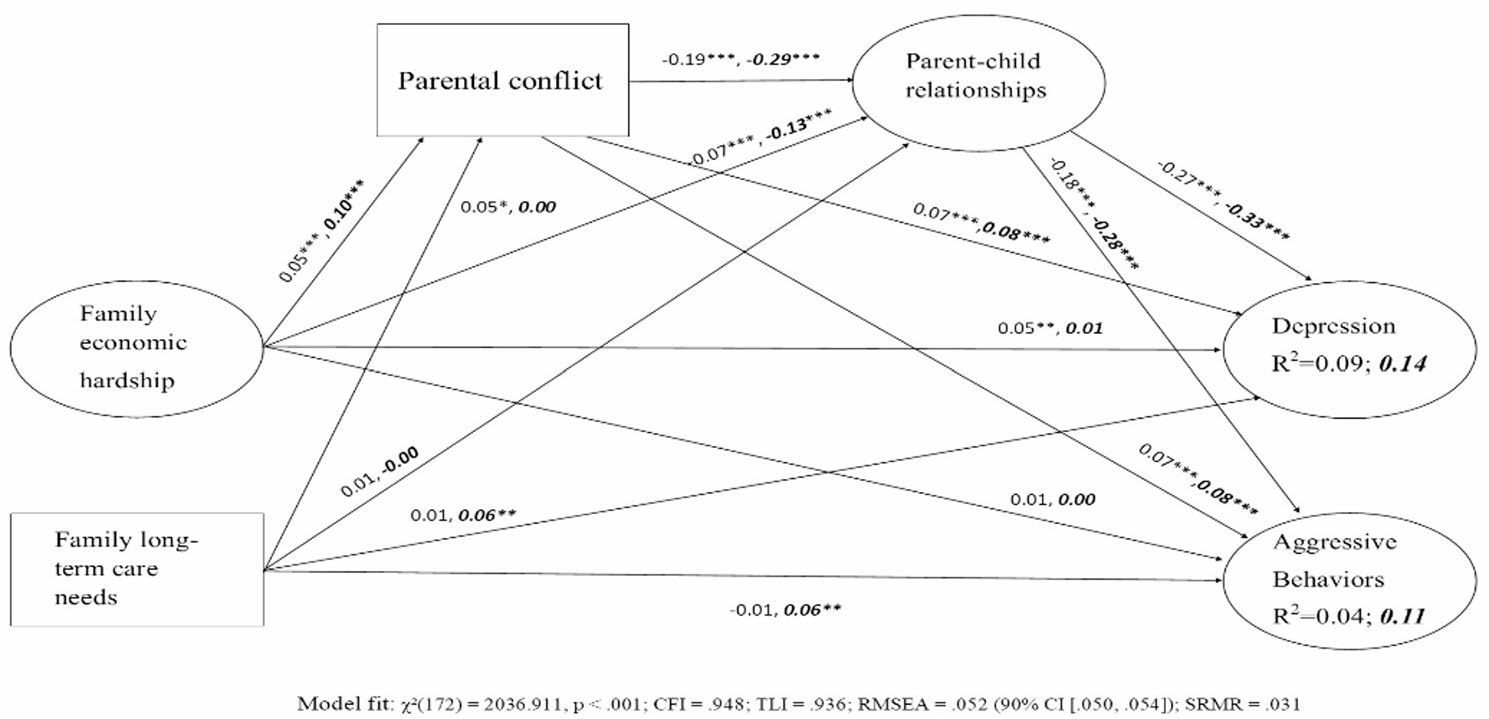
The serial longitudinal mediation model by gender *p < .05, **p < .01, ***p < .001. The coefficients in regular print and those in bold italics, represent for the results of male and female adolescents respectively

In contrast, family economic hardship did not have a significant direct association with adolescent aggressive behavior (β = 0.01, 95% CI [-0.02, 0.04], *p* > .05). The total effects from family economic hardship to aggressive behavior is 0.01 (95% CI [-0.02, 0.04], *p* > .05). It, however, showed significant indirect effects through family relational processes. Specifically, economic hardship was related to increased parental conflict (β = 0.08, 95% CI [0.05, 0.11], *p* < .001), which predicted lower parent–child relationship quality (β = −0.23, 95% CI [-0.26, − 0.20], *p* < .001) and, in turn, associated with higher aggressive behavior (β = −0.22, 95% CI [-0.25, − 0.19], *p* < .001). Indirect effects emerged through single mediation via parental conflict (β = 0.004, 95% CI [0.01, 0.02], *p* < .01), parent–child relationship (β = 0.02, 95% CI [0.03, 0.07], *p* < .001), and the sequential effects involving both mediators (β = 0.004, 95% CI [0.01, 0.01], *p* < .001), yielding a total indirect effect of β = 0.03 (95% CI [0.05, 0.09], *p* < .001). Taken together, the total effect from family economic hardship to depression through parental conflict and parent-child relationship was 0.04 (95% CI [0.05, 0.10], *p* < .05). (Fig. 2).

Similarly, family long-term care needs at Wave 1 were also directly associated with adolescent depression at Wave 2 (β = 0.03, 95% CI [0.01, 0.06], *p* < .01), and additional indirect pathways emerged through family relational mechanisms. Long-term care needs were associated with increased parental conflict (β = 0.03, 95% CI [0.00, 0.06], *p* < .05), which contributed to reduced parent–child relationship quality (β = −0.23, 95% CI [-0.26, -0.20], *p* < .001) and, in turn, increased depressive symptoms (β = −0.29, 95% CI [-0.33, − 0.26], *p* < .001). The specific single mediation effects via parental conflict (β = 0.002, 95% CI [0.00, 0.01], *p* < .05) and the sequential mediation effects through both parental conflict and parent–child relationship were statistically significant (β = 0.002, 95% CI [0.00, 0.00], *p* < .05). However, the mediation effects through parent-child relationship (β = − 0.003, 95% CI [-0.01, 0.01], *p* > .05) and total indirect effect was not significant (β = 0.001, 95% CI [-0.01, 0.01], *p* > .05). Taken together, the total effect from family long-term care needs to depression through parental conflict and parent-child relationships was 0.03 (95% CI [0.01, 0.06], *p* < .01).

In terms of adolescent aggressive behavior, family long-term care needs showed no significant direct association (β = 0.02, 95% CI [-0.01, 0.04], *p* > .05). However, long-term care needs were associated with increased parental conflict (β = 0.03, 95% CI [0.00, 0.06], *p* < .05), which contributed to reduced parent–child relationship quality (β = −0.23, 95% CI [-0.26, -0.20], *p* < .001) and, in turn, increased aggressive behaviors (β = −0.22, 95% CI [-0.25, − 0.19], *p* < .001). Specific indirect effects emerged as statistically significant, including the mediation via the parental conflict (β = 0.002, 95% CI [0.00, 0.01], *p* < .05) and the sequential pathway through both parental conflict and parent–child relationship (β = 0.001, 95% CI [0.00, 0.00], *p* < .05). Despite these significant individual effects, the mediation effects through parent-child relationship (β = − 0.002, 95% CI [-0.01, 0.00], *p* > .05) and the total indirect effect was not statistically significant (β = 0.002, 95% CI [-0.01, 0.01], *p* > .05). Taken together, the total effect of family long-term care need on aggressive behavior through parental conflict and parent-child relationships was 0.02 (95% CI [-0.01, 0.06], *p* > .05)..

Overall, the model explained small to medium proportions of variance (10.4% for depression, 6.1% for aggression), suggesting additional unmeasured factors may affect these outcomes.

### Cross-gender comparison

The multi-group analysis was conducted to examine the gender differences in Family Stress Model pathways. The multi-group model demonstrated good fit to the data: χ²(172) = 2036.911, *p* < .001; CFI = 0.948; TLI = 0.936; RMSEA = 0.052 (90% CI [0.050, 0.054]); SRMR = 0.031.

The results demonstrated that the FSM processes were consistent across gender groups, although several important differences emerged in specific pathways. The association between economic hardship and parental conflict was significantly stronger for females (β = 0.10, 95% CI [0.06, 0.14], *p* < .001) than for males (β = 0.05, 95% CI [0.01, 0.09], *p* < .05). The direct effects of long-term care needs on aggressive behaviors showed a significant gender difference with females demonstrating a positive association (β = 0.06, 95% CI [0.02, 0.11], *p* < .01) while males showed no significant direct effect (β = − 0.01, 95% CI [-0.05, 0.02], *p* > .05). The pathway from parental conflict to parent-child relationship showed a significant negative association among both genders but a stronger effect for females (β = − 0.29, 95% CI [-0.33, − 0.24], *p* < .001) compared to males (β = − 0.19, 95% CI [-0.23, − 0.15], *p* < .001). The association between parent-child relationship and depression also showed significant effects for both groups, but females demonstrated a significantly stronger association (β = − 0.33, 95% CI [-0.38, − 0.28], *p* < .001) compared to males (β = − 0.27, 95% CI [-0.31, − 0.23], *p* < .001).

Overall, these results suggested that family stressors and relational processes account for a greater proportion of variance in adolescent outcomes among females than males. For depression, the overall model explained 13.6% of the variance for females (R² = 0.136) compared to 9.2% for males (R² = 0.092). For aggressive behaviors, the model explained 10.8% of the variance for females (R² = 0.108) and 4.4% for males (R² = 0.044).

## Discussion

### Overall model

Research on the FSM has primarily focused on family economic hardships in Western contexts [8–12]. Relatively few studies have explored the influence of other contextual family stressors on adolescent development in Asian cultural settings. Using two-wave national representative data in a Chinese context, this study employed structural equation modeling to explore how family economic hardship and long-term care needs influence adolescent depression and aggressive behavior through parental conflict and parent–child relationship quality.

The results of examining the impacts of family economic hardship on adolescent development supported the FSM. The findings indicated that consistent with the theoretical framework hypothesized by the FSM, economic hardship in Wave 1 predicted adolescent depression and aggressive behaviors in Wave 2 through family relational mechanisms. Specifically, families experiencing greater economic hardship showed increased levels of parental conflict, which subsequently harmed the quality of parent–child relationships and, in turn, increased depressive symptoms and aggressive behaviors among adolescents. The mediation effects observed for economic hardship in this study aligned with patterns reported in Western samples [7, 56], suggesting that these processes may operate similarly across cultural contexts.

The findings related to family long-term care needs partially support the FSM. Family long-term care needs at Wave 1 increased parental conflict, which in turn predicted higher levels of adolescent depression and aggressive behavior at Wave 2. These results support the proposed indirect pathway through parental conflict as a mediating mechanism. However, the full serial mediation pathway was not fully supported, as family long-term care needs had a limited impact on parent–child relationships. These findings may be partly explained by the structural features of the Chinese educational system and adolescents’ strong orientation toward school life. In many regions of China, middle school students spend the majority of their day engaged in academic activities, with school schedules often extending into the late afternoon or evening, including supplementary lessons and organized after-school programs [57, 58]. This school-centered structure may reduce the amount of time adolescents spend at home, thereby limiting their exposure to or involvement in family caregiving dynamics. As a result, the impact of family long-term care needs on adolescents’ relational experiences, particularly with parents, may be attenuated.

In addition, this model accounted for only a modest proportion of variance in adolescent depression and aggressive behaviors in the Chinese context, which is relatively lower than what has been reported in many studies conducted in Western populations [11, 59]. This relatively lower explanatory power may reflect unmeasured mediators, such as parenting beliefs and parenting styles, which may interact with conflict and relationship quality to influence youth adjustment [60, 61]. The absence of these constructs in the current model may restrict its ability to account for the full range of family-level influences on adolescent outcomes. Another possible explanation might be the cultural interpretation of family-related stressors, which may shape how adolescents experience and respond to these challenges. In societies influenced by strong familism values, such as China, responsibilities like supporting disabled family members and senior family members with chronic illness are often seen as normative obligations [46] rather than exceptional stressors. These culturally embedded expectations may reduce the subjective burden of such experiences, thereby weakening their psychological impact. Ecological models of development emphasize that cultural meanings attached to stressors play a critical role in modulating their effects on individual outcomes [62], which may explain the weaker associations observed in this study.

### Gender comparison

The multi-group analysis revealed that while the proposed model demonstrated applicability across gender groups in Chinese contexts, important gender differences emerged in specific pathways. The finding that economic hardship had stronger effects on parental conflict for families with female adolescents may reflect gendered socialization patterns in Chinese culture. Traditional gender roles may make girls more aware of or responsive to family financial discussions and tensions, potentially making them more sensitive to the interpersonal consequences of economic stress [18, 49]. Similarly, the stronger association between parental conflict and parent-child relationship as well as the stronger relationship between parent-child relationship and depression among females aligns with literature suggesting that girls show greater relational orientation and vulnerability to interpersonal stressors during adolescence [48, 60, 63, 64].

The pattern where long-term care needs showed direct effects on outcomes only for females is particularly noteworthy given Chinese cultural expectations around caregiving. While both boys and girls may be exposed to family caregiving responsibilities, traditional gender socialization may make girls more directly involved in or emotionally connected to care provision, leading to stronger direct impacts on their mental and behavioral health [39, 50]. This finding suggests that in collectivist cultures where family caregiving represents normative obligations, the psychological impact may still vary by gender despite shared cultural values [46].

### Implications

While the FSM showed good applicability to economic hardship, its partial support for long-term care needs suggests that not all stressors influence family processes in the same way. Economic hardship showed consistent negative effects on family functioning across cultural contexts, while caregiving-related stress may be more contextually dependent and culturally moderated. Therefore, policies that support families facing financial strain, including income support, affordable childcare, and access to mental health resources, may have widespread and culturally transferable benefits [65, 66]. For long-term caregiving families, support services should be culturally responsive and designed to meet the unique needs and expectations of those assuming long-term care responsibilities [67]. Interventions that are both stressor-specific and context-sensitive are more likely to promote resilient family functioning and positive adolescent outcomes.

Additionally, in some family contexts, especially where maintaining family cohesion is deeply valued, there may be a tendency to prioritize outward family stability over resolving marital disagreements [68, 69]. However, in the present study, parental conflict emerged as a more consistent mediator than the parent–child relationship. Unsolved parental conflict predicted a higher risk of mental and behavioral health problems, even when parenting roles are fulfilled. This finding carries important implications for family-based interventions, suggesting that focusing solely on improving the parent–child bond may not be sufficient for adolescent mental and behavioral health if conflict between parents remains [48, 64, 70, 71].

In addition, given the findings indicating differences between genders in certain paths from family stressors to their outcomes in this study, interventions aiming at reducing depression and aggressive behavior among adolescents may consider addressing the different needs of boys and girls. For example, for female adolescents, who showed greater sensitivity to family stress processes, interventions should focus on strengthening family relational mechanisms and building resilience to interpersonal stressors. This may include programs that enhance emotional regulation in response to family conflict, develop coping strategies for family caregiving stress, and promote healthy family communication patterns. Such efforts could help mitigate the stronger pathway from family stressors to adjustment problems, especially for female adolescents. For male adolescents, while family stress processes were less influential, interventions may explore and address alternative pathways to adjustment problems that were not captured in the current model.

### Limitations

Several limitations should be acknowledged when interpreting the findings of this study. First, while the study employed a longitudinal design with two waves, the temporal intervals may not have been optimal for capturing the full development of family stress processes. Future studies should consider extending follow-up periods to better understand the temporal dynamics of these relationships. Second, this study, which is based on a secondary data analysis using the CEPS survey, is limited by the existing measures that may affect the interpretation of findings. For instance, the measures of parental conflict and family financial status are subjective and open to interpretation. In addition, the single binary question measuring long-term care needs may not adequately capture the complexity of caregiving responsibilities. Future studies should employ more objective frequency-based measures of parental conflict, such as recording specific occurrences on a weekly or monthly basis and relatively objective measures of family financial status, such as monthly income or annual income. Furthermore, the measure of family caregiving burden did not identify the specific relationship of the person requiring long-term care to the adolescent. Future research should collect more detailed information about care recipient relationships and comprehensive assessments of caregiving dimensions including type, severity, and duration are also necessary to provide a clearer understanding of family dynamics. Third, although this study utilized multi-informant data, including parent-reported financial stressors and student-reported parental conflict, depression and aggression, social desirability bias may still affect reporting of these sensitive topics. Future research should employ observational measures to evaluate the potential social desirability bias. Fourth, the study’s focus on family relational processes as mediators excluded potential mediators, such as parenting styles, parental coping, or parental mental health, which should be explored in future research.

## Data Availability

The present analyses were based on the China Education Panel Survey (CEPS) collected in China in 2013–2014 and 2014-2015 academic year. Data and codebook are available from http://ceps.ruc.edu.cn/English/Home.htm.
